# Demographic and Prognostic Factors of the Columnar Cell Variant of Papillary Thyroid Carcinoma: A National Cancer Database Study

**DOI:** 10.7759/cureus.66913

**Published:** 2024-08-15

**Authors:** Suraj Puvvadi, Nisha Reddy, Rania Jundi, Amber Chang, Peter T Silberstein, Beau Hsia

**Affiliations:** 1 College of Health Solutions, Arizona State University, Phoenix, USA; 2 College of Liberal Arts and Sciences, Arizona State University, Tempe, USA; 3 College of Biological Sciences, University of California, Davis, USA; 4 Department of Oncology, Creighton University School of Medicine, Omaha, USA; 5 Department of Oncology, Creighton University School of Medicine, Phoenix, USA

**Keywords:** rare cancer, columnar cell, papillary thyroid carcinoma, demographic factors, prognostic factors, ncdb

## Abstract

Objective

The columnar cell variant of papillary thyroid carcinoma (PTC-CC) is a rare, malignant tumor of the thyroid gland. This study uses the National Cancer Database (NCDB) to analyze demographic and prognostic factors affecting the overall survival rates of PTC-CC.

Methods

From 2004 to 2020, 7,079 patients diagnosed with columnar cell papillary thyroid carcinoma were identified in the NCDB. Patient demographics were reviewed based on categories listed in the NCDB participant user file data dictionary. Kaplan-Meier curves, log-rank tests, and multivariable Cox hazard regression models were used to analyze the significance of demographic and prognostic factors on overall survival rates of PTC-CC.

Results

Multivariate analysis demonstrated each five-year increment in age was associated with a 30% increase in mortality (hazard ratio (HR) = 1.30, 95% confidence interval (CI): 1.25-1.36, P < 0.001). Charlson-Deyo scores displayed similar incremental increases, such that patients with a score ≥ 3 had a 154% increase in mortality risk relative to a score of 0 (HR = 2.54; 95% CI: 1.75-3.68, P < 0.001). Black individuals had a 70% increase in mortality compared to White individuals (HR = 1.70, 95% CI: 1.25-2.30, P < 0.001), while all Other races had the highest 10-year survival rate of 92.7%. Females had a significant 37% decrease in mortality compared to males (HR = 0.63, 95% CI: 0.54-0.73, P < 0.001). Patients in the lowest income quartiles were found to have a significant increase in mortality compared to the highest income group (HR = 0.54; 95% CI: 0.41-0.71, P < 0.001). Survival rates were negatively correlated with NCDB Analytic Staging increases.

Conclusion

In general, age, sex, race, education, income, comorbidities, and cancer staging were found to be predictive factors of overall survival rates of PTC-CC. However, insurance status and education levels did not result in significant differences.

## Introduction

Papillary thyroid carcinoma (PTC) is a malignant, epithelial tumor accounting for 90% of all thyroid cancers [1]. The columnar cell (CC) variant is a highly rare and aggressive subtype, accounting for only 0.15-0.20% of all PTCs [2]. Papillary thyroid carcinoma, columnar cell (PTC-CC) is histologically defined to have papillae lined with stratified-nuclear columnar cells and structurally defined to exhibit hypercellularity and lack of nuclear pseudoinclusions [3]. Compared to typical PTC, the CC variant typically has higher rates of nodal and distant metastases and lower survival rates [4].

A 1998 clinicopathologic study indicates PTC-CC as being a morphologically distinct but not clinically distinct subtype of PTC [5]. A 2019 Surveillance, Epidemiology, and End Results (SEER) study found that the average age at diagnosis for PTC-CC varies but has a median of about 53 years, with females having a disproportionately greater incidence rate (71.9%) than males (28.1%) [6]. Surgically, an overwhelming majority of cases (89.5%) conducted a total thyroidectomy. Overall, the period of survival post-diagnosis was reported to be around 44 ± 33 months [6].

Treatment procedures for PTC-CC depend on the extent of tumor size and metastasis. Surgical resection of the thyroid by a lobectomy or thyroidectomy is common and is sometimes supplemented with radioactive iodine therapy and standard chemotherapy or radiotherapy [5].

PTC-CC can manifest as either a highly aggressive or a relatively indolent tumor. The factors that typically influence its manifestation revolve around tumor size, circumscription, and encapsulation [7]. While there is currently no specific survival rate data on PTC-CC, PTC presents a 10-year survival rate of 93%. However, the aforementioned SEER study’s univariate Cox regression analysis found Black individuals (referred to as “African origin” in the original study), male sex, and tumor extension to be major risk factors, and surgical resection and radiation therapy to be major protective factors [4].

The goal of our study is to understand the demographic and prognostic factors for PTC-CC. In this paper, we explain the association between overall survival of PTC-CC variant patients and age, sex, race, income, education, insurance status, NCDB Analytic Stage, adjuvant chemotherapy, adjuvant radiation, and Charlson-Deyo comorbidity score. Through providing novel analytics, we aim to evaluate the relationship between demographic and prognostic factors and their association with patient survival rates of the columnar cell variant of papillary thyroid carcinoma.

## Materials and methods

This is a retrospective cohort study of patients diagnosed with papillary thyroid carcinoma from 2004 to 2020. The study was conducted at Creighton University Health Sciences Campus, Phoenix, United States. Patients were identified from the National Cancer Database (NCDB). NCDB is sponsored by the American College of Surgeons and the American Cancer Society. It contains data from over 1,500 Commission on cancer-accredited facilities and includes over 70% of new cancer diagnoses in the United States. Authors were granted access to de-identified patient data from the NCDB via the participant user data files program. The NCDB data is stored on a local and internal computer drive that is encrypted with hardware keys to provide advanced levels of security.

This study had inclusion/exclusion criteria that patients had to meet in order to participate in the study. Patients with PTC were recognized from NCDB data utilizing ICD-O-3 codes, columnar cell variant 8344, and PTC variants 8050, 8260, 8342, and 8343. Patients were excluded from the cohort if they had concurrent tumors or had any missing clinical or demographic factors. All patients met the inclusion/exclusion criteria for the final study cohort.

Patients were analyzed by age, sex, race, income, education, insurance status, tumor size, NCDB Analytic Stage, primary anatomic site, surgery, adjuvant and neoadjuvant chemotherapy, radiation, distance traveled for healthcare, and Charlson-Deyo comorbidity score. The study categorizes race into White, Black, and Other groups. The race group classified as Other incorporates American Indian, Aleutian or Eskimo, Chinese, Japanese, Filipino, Hawaiian, Korean, Vietnamese, Kampuchean, Asian Indian or Pakistani not otherwise specified (NOS), Asian Indian, Micronesian NOS, Other Asian, Asian NOS, Oriental NOS, and Pacific Islander NOS populations. The income category in the study measures the median household income from 2016 to 2020 using the zip codes patients provided at the time of diagnosis. The education category was studied by the percentage of residents within the patient's zip code who did not graduate from high school between 2016 and 2020. The variable NCDB Analytic Stage includes the pathologic staging when obtainable; if unavailable, clinical staging is utilized. To study a patient's insurance status, researchers categorized it into five groups: uninsured, private, Medicare, Medicaid, and other government insurance. Distance traveled for health care was calculated as the number of miles between the patient's home and the hospital that documented the case. The Charlson-Deyo score measured comorbidities, and the patients were divided into groups with scores of 0, 1, 2, and ≥ 3.

To calculate the median overall survival at two, five, and 10 years between the variables of interest, Kaplan-Meier curves were plotted and the survival tables were used. A multivariable Cox hazard regression model was utilized to specify independent prognostic factors. Variables contained in the multivariable Cox model were age, sex, race, income, education, insurance status, tumor size, NCDB Analytic Stage, primary anatomic site, neoadjuvant chemotherapy, neoadjuvant radiation, distance traveled for health care, and Charlson-Deyo comorbidity score. For the multivariable Cox model, five-year increments were used for age, 10-mm increments were used for tumor size, and 50-mile increments were used for distance traveled for health care. We used 25-year increments for the Kaplan-Meier survival curve based on age. We accounted for the correlation of patients within the same facility with a robust sandwich covariance matrix. The functional form of continuous variables was measured with Locally Estimated Scatterplot Smoothing (LOESS) methods. The symmetrical hazards assumption for each variable was analyzed with log-negative-log survival curves and statistical interaction with time.

Descriptive statistics and unadjusted survival analysis were operated using IBM Statistical Package for the Social Sciences (SPSS) for Macintosh, Version 29 (Released 2023 by IBM Corp., Armonk, New York, United States). P < 0.05 indicates statistical significance.

## Results

Patient characteristics

Descriptive statistics for this cohort of patients are displayed in Tables 1-3. There were a total of 7,079 patients identified in the database from 2004 to 2020 with columnar cell variants of papillary carcinoma. There was a female predominance (72.6%, N = 5136), with the majority of individuals being White individuals (81.3%, N = 5754). The median age of diagnosis was 52 years (mean = 52, range = 0-90, SD = 16.35). Most of the patients were privately insured (66.6%, N = 4712), and the second most common insurance plan was Medicare (23.1%, N = 1637). In regards to education levels, individuals were spread out rather evenly across four quartiles, with the highest percentage of patients in the third most educated group (28.6%, N = 2025). For income brackets, the largest median household quartile percentage (52.2%, N = 3695) was for the highest income group ($74,063 or more). Most patients were classified as free from co-morbidities, with 82.4% (N = 5834) having a Charlson-Deyo score of 0. The most common primary site was the thyroid gland (99.9%, N = 7071), and the median tumor size at diagnosis was 19 mm. About half of the patients in the cohort (53.2%, N = 3766) were designated as NCDB Analytic Stage I. An overwhelming majority of individuals (99.4%, N = 7040) were surgically treated at their primary site. The most common additional treatment was radiation (62.9%, N = 4454), followed by adjuvant hormone therapy (56.6%, N = 4005). Adjuvant chemotherapy treatment was less common (0.9%, N = 65). With respect to margins following procedural intervention, 72.7% (N = 5144) of the patients had no residual tumor left. The rest of the patients had microscopic, macroscopic, or non-evaluable margins, or they were otherwise not treated with primary site surgery.

**Table 1:** Clinical and demographic characteristics of patients with PTC-CC (N = 7,079) PTC-CC: papillary thyroid carcinoma-columnar cell

Variable	N = 7,079	% of total
Sex		
Male	1943	27.4
Female	5136	72.6
Race		
White	5754	81.3
Black	408	5.8
Other	917	13
Age (years)		
Mean ± Standard deviation	51.72 ± 16.351	
Median (interquartile range)	52.00 (39.5-64.5)	
Zip code-level median household income (2016-2020, $)		
< $46,277	782	11
$46,277-$57,856	1117	15.8
$57,857-$74,062	1485	21
≥ $74,063	3695	52.2
Zip code-level education (% without high-school degree, 2020)		
≥ 15.3%	1394	19.7
9.1%-15.2%	1775	25.1
5%-9%	2025	28.6
< 5%	1885	26.6
Insurance status		
Uninsured	135	1.9
Private	4712	66.6
Medicaid	545	7.7
Medicare	1637	23.1
Other government	50	0.7
Distance traveled for health care (miles)		
Mean ± Standard deviation	25.658 ± 77.8258	
Median (interquartile range)	11.9 (2.3-21.5)	
Charlson-Deyo comorbidity score		
0	5834	82.4
1	976	13.8
2	174	2.5
≥ 3	95	1.3

**Table 2:** Tumor characteristics of patients with PTC-CC (N = 7,079) NCDB: National Cancer Database; PTC-CC: papillary thyroid carcinoma-columnar cell

Variable	N = 7,079	% of total
Tumor size (mm)		
Mean ± Standard deviation	24.0365 ± 24.721	
Median (interquartile range)	19 (10-28)	
NCDB Analytic Stage		
I	3766	53.2
II	634	9
III	1558	22
IV	1121	15.8

**Table 3:** Treatment characteristics of patients with PTC-CC (N = 7,079) NOS: not otherwise specified; PTC-CC: papillary thyroid carcinoma-columnar cell

Variable	N = 7,079	% of total
Adjuvant therapy		
Received adjuvant chemotherapy	65	0.9
Received adjuvant radiation	4454	62.9
Surgical margins		
No residual tumor	5144	72.7
Residual tumor, NOS	542	7.7
Microscopic residual tumor	1083	15.3
Macroscopic residual tumor	83	1.2

Survival analysis

The overall survival for the cohort is displayed in Figure 1. Survival by specific variables is shown in Figures 2-10. Table 4 shows two, five, and 10 year survival estimates by variable. Multivariable Cox model results are in Table 5.

**Figure 1:**
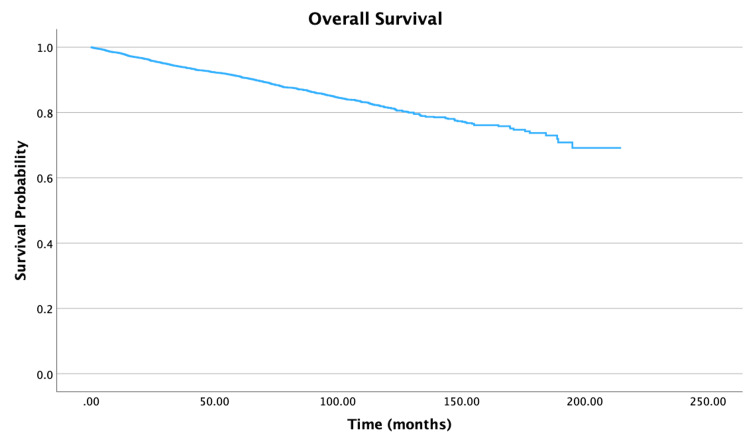
Overall survival of patients with PTC-CC (N = 7,079) PTC-CC: papillary thyroid carcinoma-columnar cell

**Figure 2:**
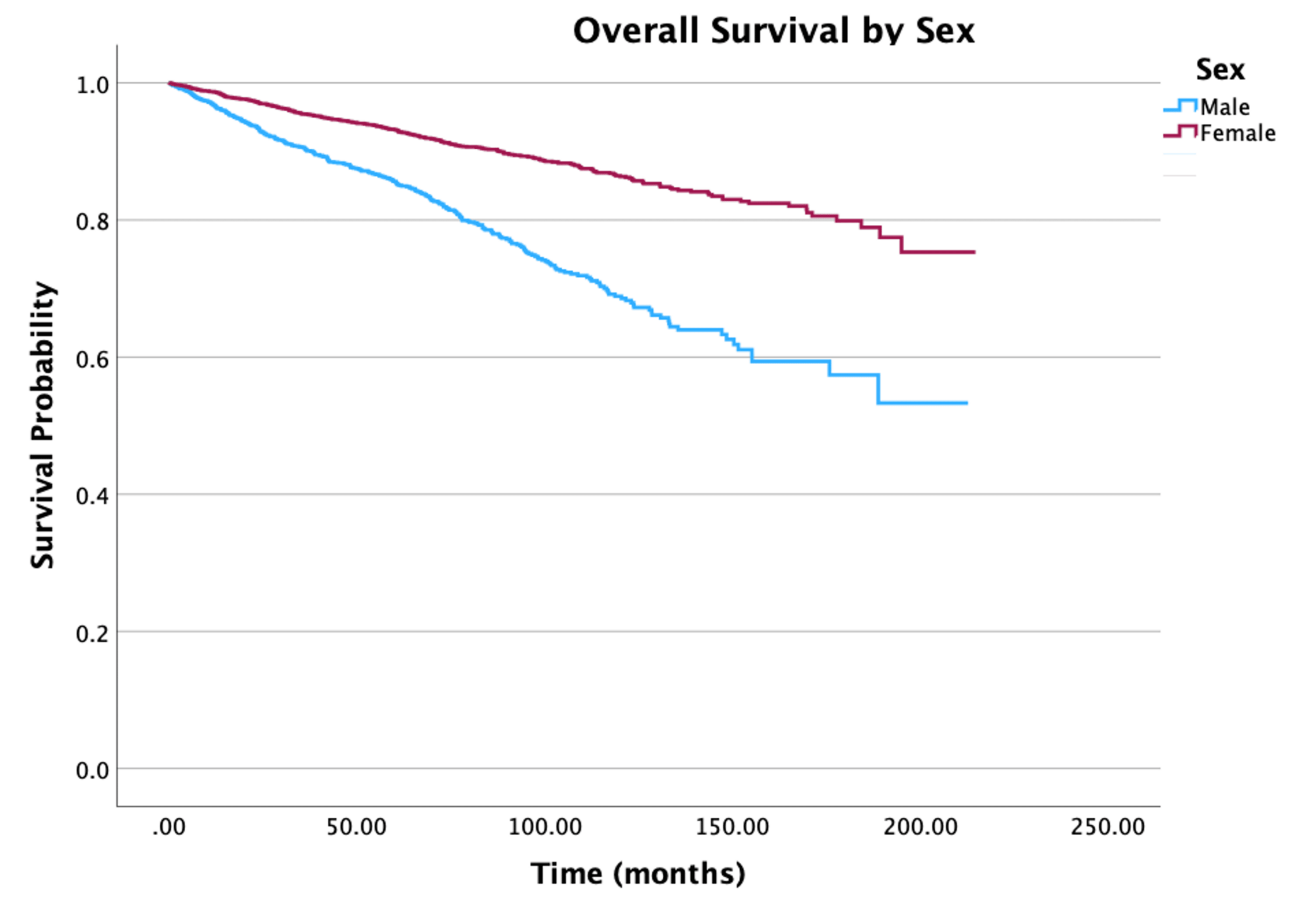
Overall survival by sex of patients with PTC-CC (N = 7,079, P < 0.001) PTC-CC: papillary thyroid carcinoma-columnar cell

**Figure 3:**
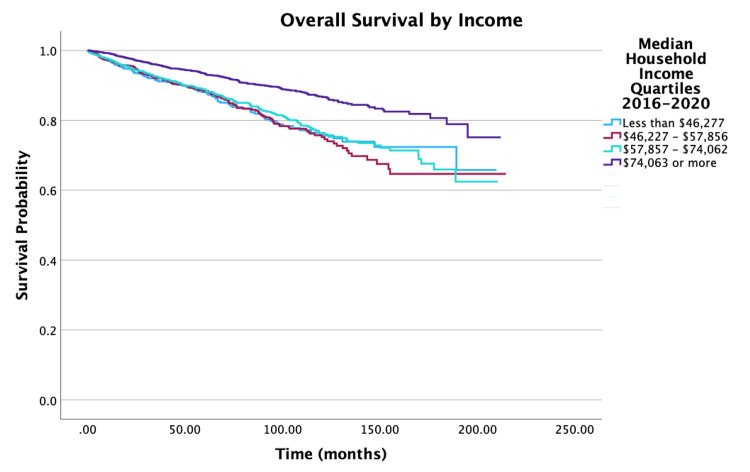
Overall survival by income of patients with PTC-CC (N = 7,079, P < 0.001) PTC-CC: papillary thyroid carcinoma-columnar cell

**Figure 4:**
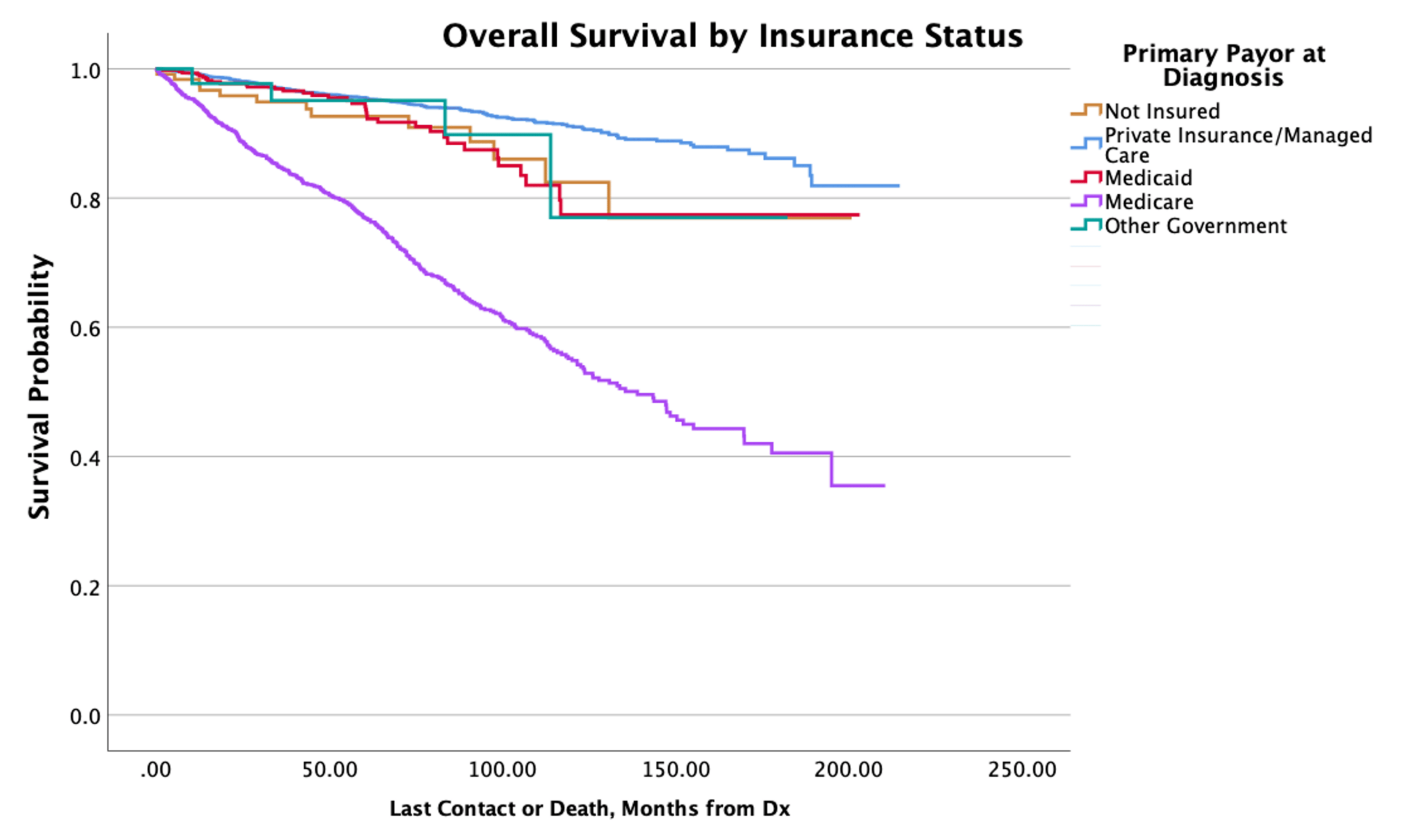
Overall survival by insurance status of patients with PTC-CC (N = 7,079, P < 0.001) PTC-CC: papillary thyroid carcinoma-columnar cell

**Figure 5:**
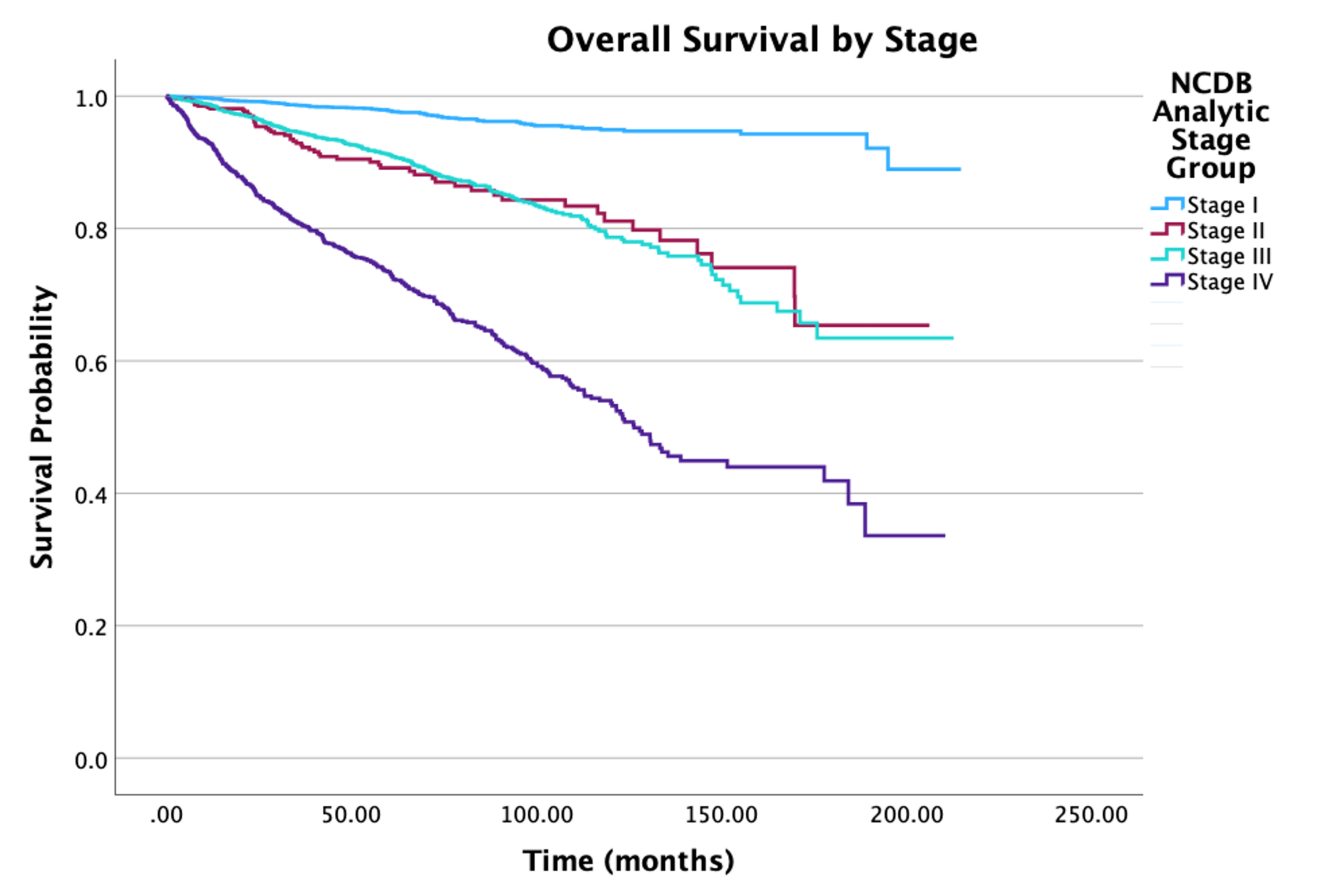
Overall survival by NCDB Analytic Stage of patients with PTC-CC (N = 7,079, P < 0.001) NCDB: National Cancer Database; PTC-CC: papillary thyroid carcinoma-columnar cell

**Figure 6:**
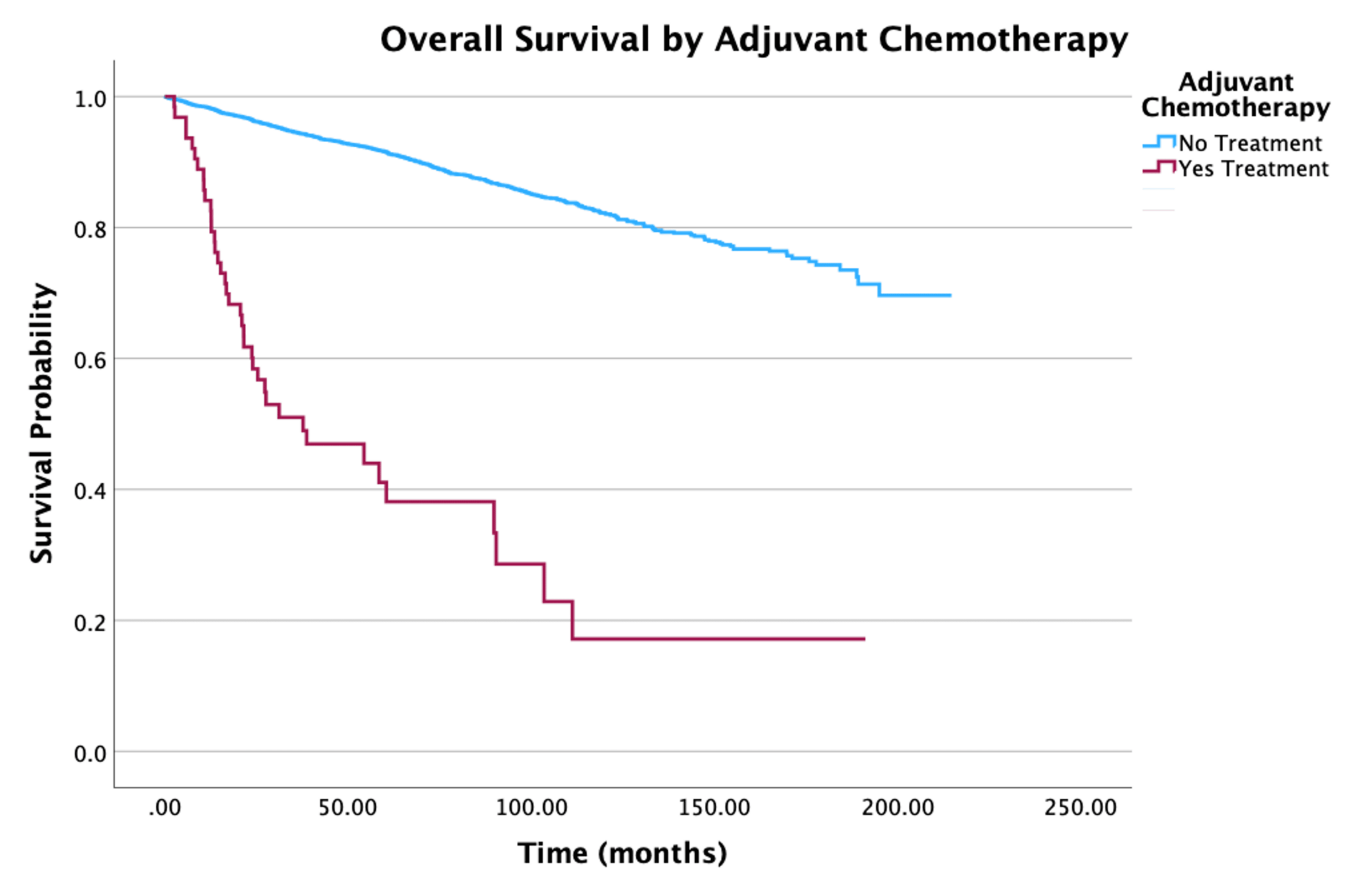
Overall survival by adjuvant chemotherapy of patients with PTC-CC (N = 7,079, P < 0.001) PTC-CC: papillary thyroid carcinoma-columnar cell

**Figure 7:**
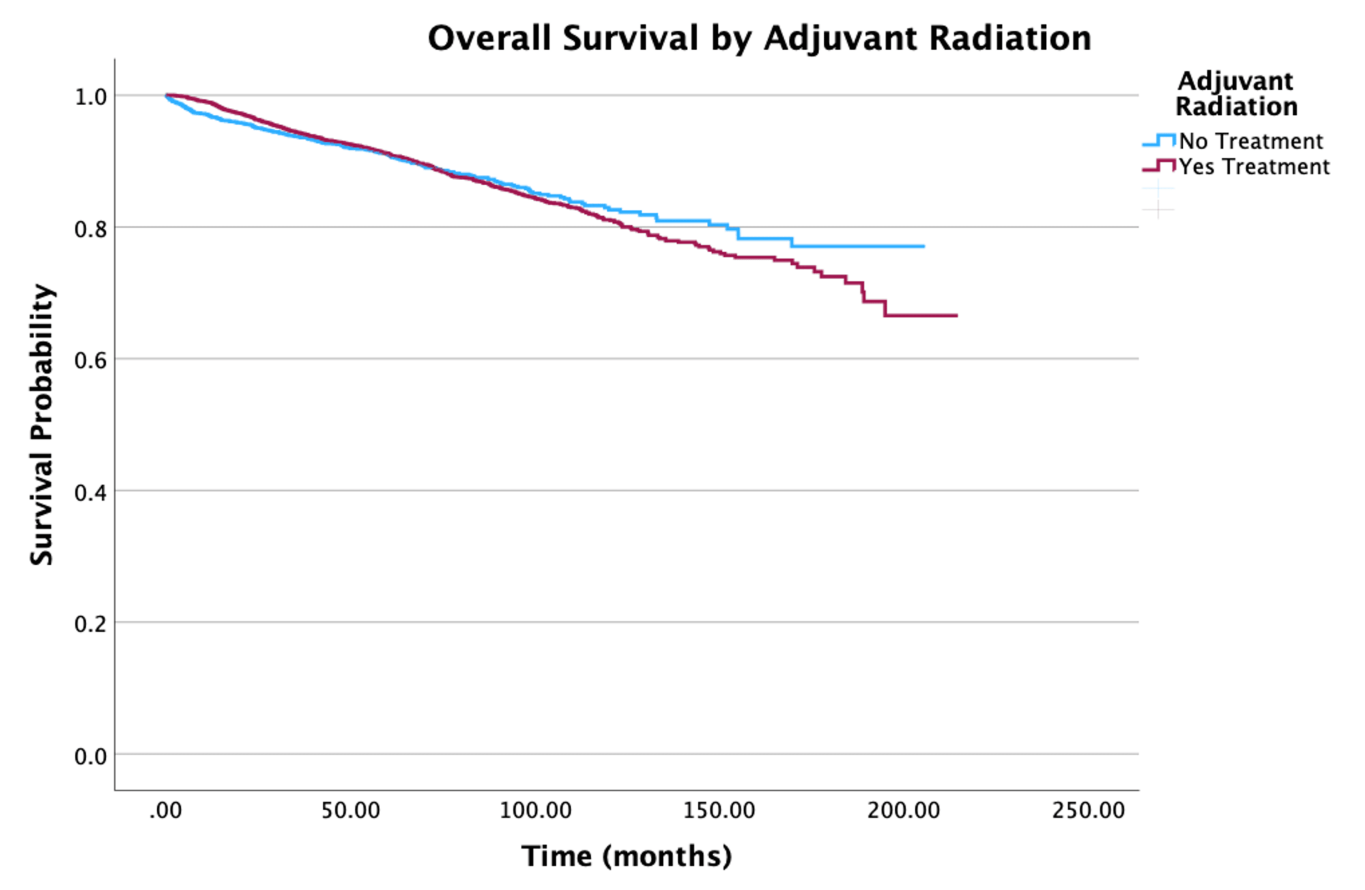
Overall survival by adjuvant radiation therapy of patients with PTC-CC (N = 7,079, P = 0.724) PTC-CC: papillary thyroid carcinoma-columnar cell

**Figure 8:**
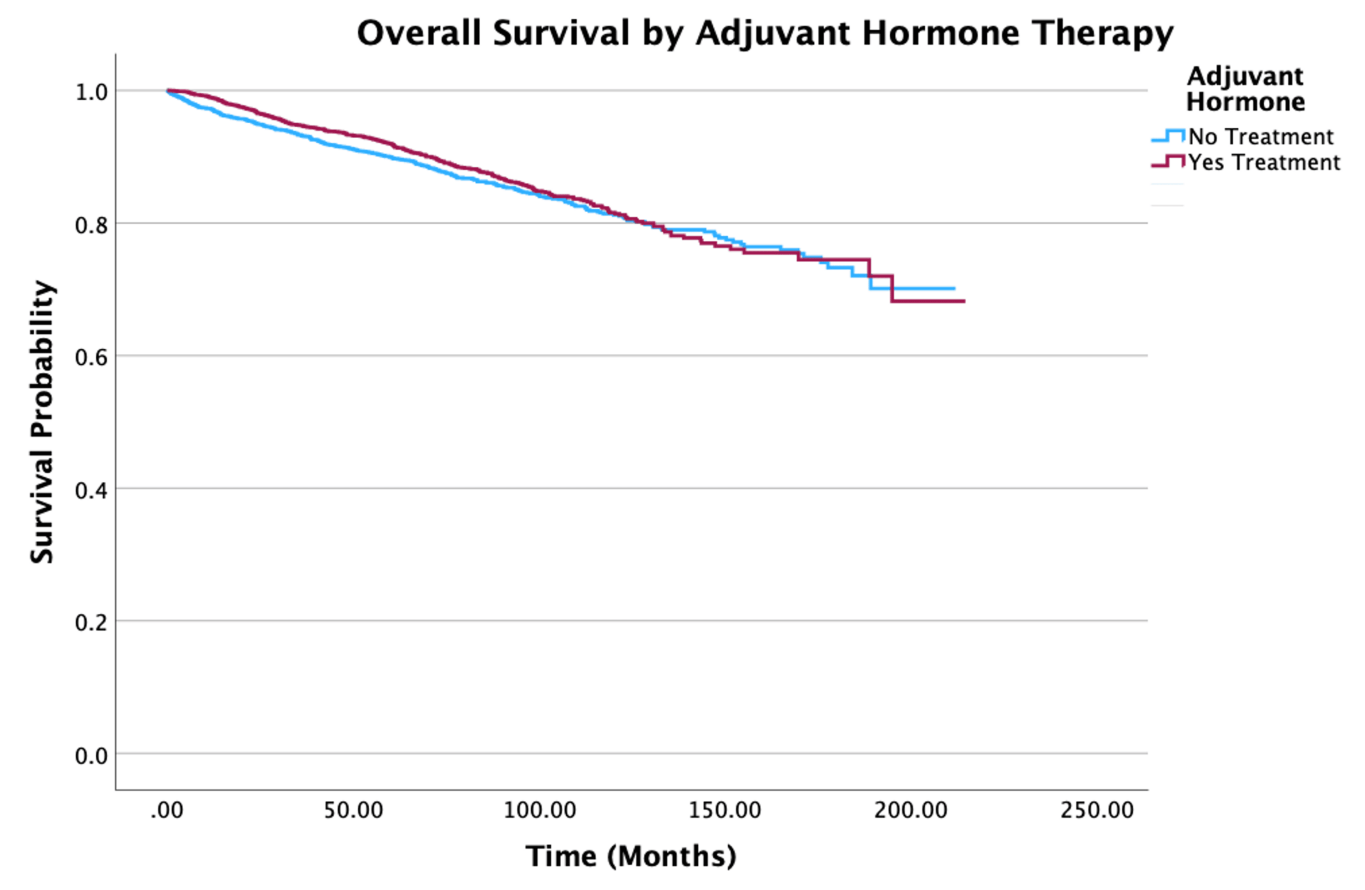
Overall survival by adjuvant hormone therapy of patients with PTC-CC (N = 7,079, P = 0.107) PTC-CC: papillary thyroid carcinoma-columnar cell

**Figure 9:**
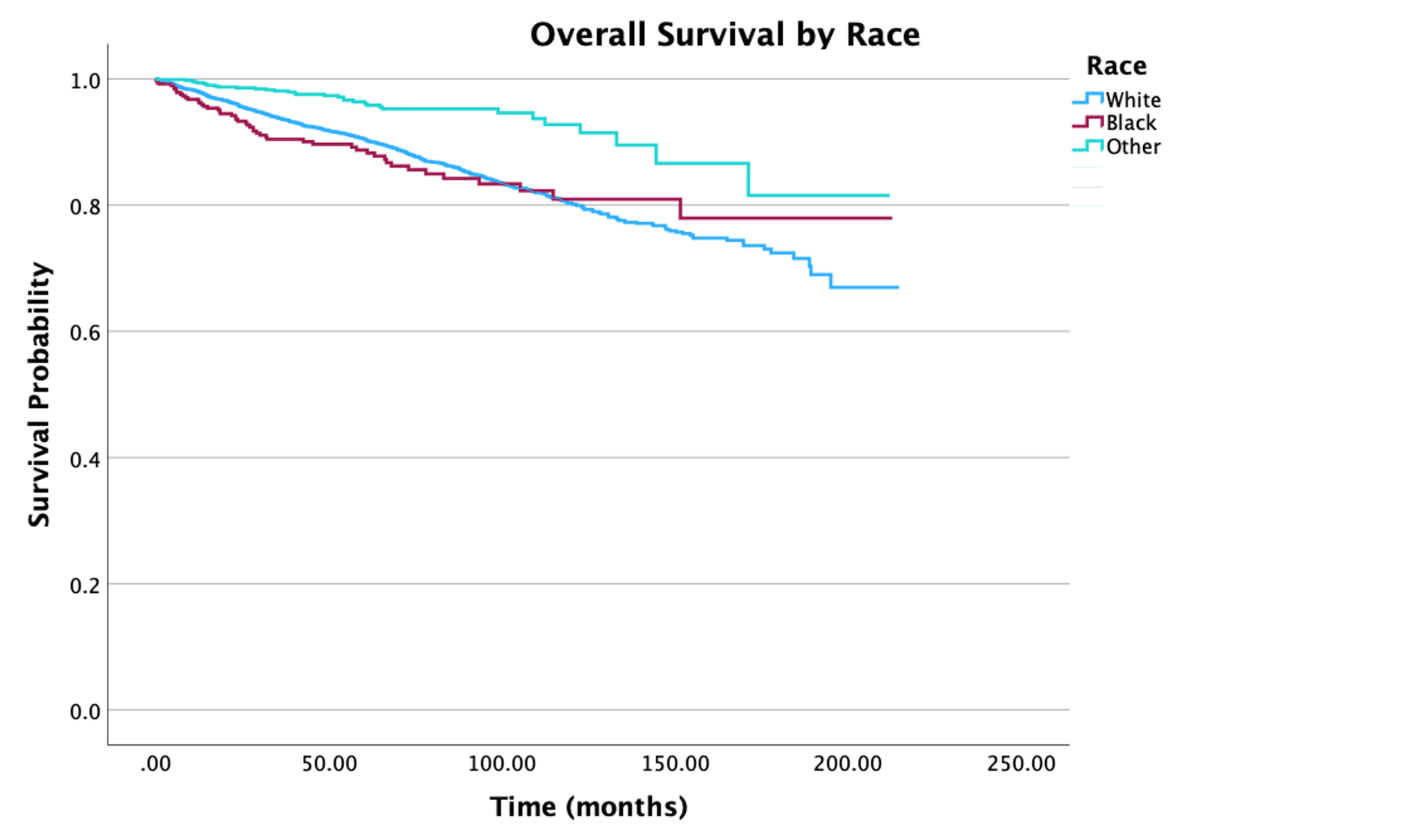
Overall survival by race of patients with PTC-CC (N = 7,079, P < 0.001) PTC-CC: papillary thyroid carcinoma-columnar cell

**Figure 10:**
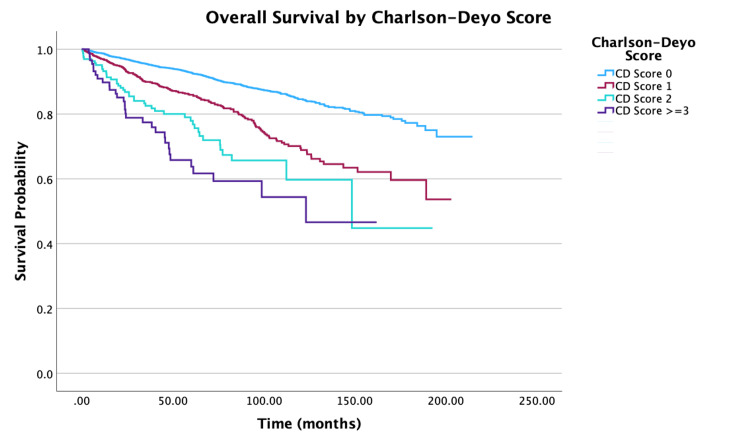
Overall survival by Charlson-Deyo score of patients with PTC-CC (N = 7,079, P < 0.001) CD: Charlson-Deyo score; PTC-CC: papillary thyroid carcinoma-columnar cell

**Table 4:** Median two, five, and 10-year survival estimates of patients with PTC-CC (N = 7,079) NCDB: National Cancer Database; PTC-CC: papillary thyroid carcinoma-columnar cell

Variable	2-year (%)	5-year (%)	10-year (%)
Sex			
Male	93.3	85.6	68.9
Female	97.1	93.2	86.5
Race			
White	95.8	90.5	80.3
Black	93.3	88.7	80.9
Other			
Zip code-level median household income (2016-2020, $)			
< $46,277	93.7	88.1	75.7
$46,277-$57,856	95.2	88.3	75.7
$57,857-$74,062	94.6	88.9	76.4
≥ $74,063	97.3	93.4	86.8
Zip code-level education (% without high-school degree, 2020)			
≥ 15.3%	95.8	90.8	79.9
9.1%-15.2%	96.3	91.1	80.3
5%-9%	95.5	90.1	80.1
< 5%	96.5	92.4	85.2
Age (years)			
0-25	99.3	99.3	99.3
26-50	99	98.1	95.8
51-75	95	88.2	77.1
76-100	84.2	67.2	30.7
NCDB Analytic Stage			
I	99.2	97.8	94.9
II	95.8	89.2	81.1
III	96.7	91.2	78.7
IV	85.7	73.5	54

**Table 5:** Multivariable Cox regression model of patients with PTC-CC (N = 7,079) NCDB: National Cancer Database; PTC-CC: papillary thyroid carcinoma-columnar cell

Variable	HR (95% Confidence Interval)	P values
Age		
Age (5 years)	1.30 (1.25-1.36)	<0.001
Gender		
Males vs. females	0.63 (0.54-0.73)	<0.001
Race and ethnicity		
White vs. Black	1.70 (1.25-2.30)	<0.001
White vs. Other	0.57 (0.40-0.82)	0.002
Black vs. Other	0.34 (0.22-0.53)	<0.001
Charlson-Deyo score		
0 vs. 1	1.41 (1.19-1.68)	<0.001
0 vs. 2	2.12 (1.55-2.91)	<0.001
0 vs. ≥ 3	2.54 (1.75-3.68)	<0.001
1 vs. 2	1.50 (1.07-2.10)	0.019
1 vs. ≥ 3	1.79 (1.21-2.65)	0.004
2 vs. ≥ 3	1.20 (0.75-1.91)	0.453
Zip code-level median household income (2020 US Dollars)		
< $46,277 vs. $46,227-$57,856	0.86 (0.66-1.10)	0.231
< $46,277 vs. $57,857-$74,062	0.89 (0.69-1.15)	0.359
< $46,277 vs. ≥ $74,063	0.54 (0.41-0.71)	<0.001
$46,227-$57,856 vs. $57,857-$74,062	1.04 (0.84-1.28)	0.735
$46,227-$57,856 vs. ≥ $74,063	0.64 (0.51-0.79)	<0.001
$57,857-$74,062 vs. ≥ $74,063	0.61 (0.50-0.74)	<0.001
Zip code-level education (2020, % No high-school diploma)		
≥ 15.3% vs. 9.1%-15.2%	1.13 (0.90-1.41)	0.302
≥ 15.3% vs. 5.0%-9.0%	1.18 (0.93-1.50)	0.167
≥ 15.3% vs. < 5.0%	1.36 (1.04-1.78)	0.023
9.1%-15.2% vs. 5.0%-9.0%	1.05 (0.86-1.28)	0.634
9.1%-15.2% vs. < 5.0%	1.21 (0.96-1.52)	0.1
5.0%-9.0% vs. < 5.0%	1.16 (0.94-1.42)	0.166
Insurance		
None vs. Private	0.72 (0.41-1.26)	0.252
None vs. Medicaid	1.19 (0.63-2.25)	0.602
None vs. Medicare	1.02 (0.58-1.80)	0.951
None vs. Other government	0.47 (0.15-1.47)	0.194
Private vs. Medicaid	1.65 (1.15-2.36)	0.006
Private vs. Medicare	1.42 (1.15-1.74)	<0.001
Private vs. Other government	0.66 (0.24-1.79)	0.411
Medicaid vs. Medicare	0.86 (0.59-1.24)	0.418
Medicaid vs. Other government	0.40 (0.14-1.14)	0.085
Medicare vs. Other government	0.46 (0.17-1.25)	0.129
Treatment		
No adjuvant chemotherapy vs. adjuvant chemotherapy	3.91 (2.80-5.47)	<0.001
No adjuvant radiation vs. adjuvant radiation	0.83 (0.71-0.97)	0.021
No adjuvant hormone therapy vs. adjuvant hormone therapy	0.85 (0.74-0.99)	0.034
NCDB Analytic Stage		
Stage I vs. Stage II	1.66 (1.18-2.32)	0.003
Stage I vs. Stage III	1.88 (1.46-2.42)	<0.001
Stage I vs. Stage IV	4.05 (3.16-5.19)	<0.001
Stage II vs. Stage III	1.13 (0.85-1.52)	0.402
Stage II vs. Stage IV	2.45 (1.85-3.24)	<0.001
Stage III vs. Stage IV	2.16 (1.82-2.56)	<0.001

Looking at age, five- and 10-year survival rate probabilities decreased with increasing age, with the oldest group of individuals (ages 76 to 100 years) having survival rates of 67.2% and 30.7%, respectively. Multivariable analysis showed that an increase in five years of age was associated with a 30% increase in mortality (hazard ratio (HR) = 1.30, 95% confidence interval (CI): 1.25-1.36, P < 0.001). For race, patients whose race identified as Other had the highest 10-year survival rates at 92.7% compared to 80.3% for White individuals and 80.9% for Black individuals. When adjusted for all other variables, Black individuals had a 70% increase in mortality when compared to White individuals (HR = 1.70, 95% CI: 1.25-2.30, P < 0.001). For survival by sex, the unadjusted 10-year survival rate for females was higher at 86.5%, compared to males at 68.9%. After adjusting for other variables in the multivariable model, females had a 37% decrease in mortality compared to males (HR = 0.63, 95% CI: 0.54-0.73, P < 0.001). An increase in Charlson-Deyo scores is correlated with a decrease in survival rates. Multivariable analysis demonstrated that for patients with a Charlson-Deyo score of 0, patients with a score of 1 and ≥ 3 in comparison had a 41% increase (HR = 1.41; 95% CI: 1.19-1.68, P < 0.001) and 154% increase (HR = 2.54; 95% CI: 1.75-3.68, P < 0.001) in mortality risk, respectively. Analyzing education levels by the four quartile distinctions, there was a 36% increase in mortality for patients in the lower education groups compared to those in the highest education group (HR = 1.36, 95% CI: 1.04-1.78, P = 0.023). There was also a significant difference in mortality risks for patients in the lowest and highest income groups (HR = 0.54, 95% CI: 0.41-0.71, P < 0.001).

Treatment-wise, the patients who received adjuvant chemotherapy had decreased two and five-year survival rate probabilities of 58.4% and 41.1%, respectively. Multivariate analysis showed that patients who received adjuvant chemotherapy were 3.91 times more susceptible to mortality compared to patients who did not receive the treatment (HR = 3.91, 95% CI: 2.80-5.47, P < 0.001). Overall, two, five, and 10-year survival rate probabilities steadily decreased as NCDB Analytic Staging groups increased from Stage I to Stage IV. More specifically, within the staging groups, Stage II patients had a 66% increase in mortality compared to Stage I patients (HR = 1.66, 95% CI: 1.18-2.32, P = 0.003). Stage III patients had an increase of 88% in mortality relative to Stage I patients (HR = 1.88, 95% CI: 1.46-2.42, P < 0.001). Finally, Stage IV patients had a three-times higher mortality compared to Stage I patients (HR = 4.05, 95% CI: 3.16-5.19, P < 0.001).

There were no significant differences in the mortality risks for patients who were uninsured compared to those who were insured privately, with Medicaid, Medicare, or other government plans. It was noted that there were no significant differences in mortality risks when comparing the lowest group with the second and third quartile groups. For the NCDB Analytic Stages, Stage III was not associated with significant mortality differences in comparison to Stage II, and for Charlson-Deyo scores, scores of ≥ 3 were not associated with significant mortality differences compared to scores of 2. 

## Discussion

Given the rarity of PTC-CC, there is a paucity of literature characterizing its demographic and prognostic factors. This is one of the comprehensive studies to date analyzing the prognostic factors for the columnar cell variant of papillary thyroid carcinoma.

The literature shows that thyroid cancer, in general, is 2.9 times more common in women than men [8]. Similarly, for PTC-CC, multiple clinical reports indicate a female predominance, with one study noting a 70%-30% female to male ratio, which is consistent with our study at 72.6% female (N = 5136) [3]. The patient population from this same study reported an average age of 49.4, which is close to our reported average age of 52 years old [3]. Other case studies tend to report more females than males but include a more diverse age range within their study cohorts [9,10].

The most common primary site for PTC-CC is in the thyroid region, which is consistent with our findings [3]. Rates and locations of metastases vary heavily. If PTC-CC metastasizes, it will typically affect the lymph nodes first, followed by extrathyroidal extension to the muscle. In the most aggressive cases, it may extend to the lungs and brain [2].

In a study by the Clinical University Hospital, the median tumor size at diagnosis was 19 mm, which is consistent with our study [11]. While prognostic factors like tumor size and patient age are considered when evaluating the cancer’s pathway of progression, this study suggests that aggressiveness of PTC-CC is independent of tumor size and age. Instead, the literature suggests that location and prevalence of metastases and presence of the BRAFV600E mutation may be more indicative of PTC-CC than other types of thyroid cancer [11,12]. Specifically, the columnar cell variant, compared to classical variants, exhibits higher rates of extrathyroidal extension (56.3% vs. 14.7%) and lymph node metastases (56.3% vs. 26.4%). The BRAFV600E mutation indicates a more stark difference (80.0% vs. 26.4%) [11]. Given the significance of the BRAFV600E mutation, we consider gene mutation analysis of PTC-CC patients to be an important future study within this field.

Surgery is the typical treatment for papillary thyroid carcinomas of the columnar cell subtype [5]. Upon initial suspicion of PTC-CC, surgery is performed conservatively through procedures such as lobectomies or subtotal thyroidectomies. However, once the patient receives a confirmed PTC-CC diagnosis, a total thyroidectomy is conducted. Oftentimes, surgery is sufficient for complete recession of the disease; in a post-surgical survival analysis (average of 5.8 years), 81% of patients were alive with no indication of PTC-CC [5]. While complications with total thyroidectomy may occur, repeated surgeries following the complete removal of the thyroid are minimal, occurring in less than 5% of patients [13, 14]. Our study is consistent with these findings, as 99.4% (N = 7040) of the NCDB cohort underwent surgical treatment. Following surgery, radioactive therapy is often performed via radioactive iodine administration or external beam radiotherapy [13]. Radioactive therapy serves to minimize any collateral, postoperative microscopic diseases, address and prevent further differentiation of PTC-CC, and detect any PTC-CC recurrence [13]. Adjuvant radiotherapy is common within the NCDB cohort, with 62.9% (N = 4454) of patients undergoing radiation as an additional treatment. With up to 66% of patients developing hypothyroidism following total thyroidectomy, adjuvant hormonal therapy is often used to address postoperative low levels of free thyroxine (fT4) and high levels of thyroid-stimulating hormone (TSH) [15]. Levothyroxine supplementation may be used reactively to suppress TSH and increase thyroid hormone levels; it may also be used proactively to prevent PTC-CC recurrence and decrease mortality rate in high-risk patients [15]. Adjuvant hormonal therapy as an additional treatment intervention is represented within 56.6% (N = 4005) of the NCDB cohort. Total thyroidectomy with additional adjuvant therapeutics for hormone replacement or radiotherapy, based on lab results and anatomical findings, is often the treatment course for PTC-CC patients. Chemotherapy is extremely rare as a treatment, as it is reserved for patients with recurrent PTC-CC or excessive remnant disease [1]. Chemotherapy is warranted as a treatment if and only if failure of complete PTC-CC remission occurs, despite thyroidectomy and adjuvant therapies [1]. This idea is reflected within our NCDB cohort, as only 0.9% (N = 65) of patients underwent chemotherapy.

Socioeconomic status (SES) factors, such as race and educational attainment, play an important prognostic role when predicting the diagnostic and mortality rates of different cancers [16]. The role of SES in outcomes of PTC-CC has yet to be investigated in a prospective study context. However, a large-scale SEER study has been conducted to understand the risk factors for survival based on SES factors like age, sex, and race [6]. Compared to the classical (HR = 0.156) and follicular (HR = 0.452) variants, the columnar cell variant of papillary carcinoma carries a much higher risk of death [6]. Our multivariate Cox regression model revealed that females have a 37% decrease in all-cause mortality rates compared to their male counterparts. This finding aligns with the SEER study, which shows that males are 59.9% more susceptible to death than females [6]. Correlations between race and PTC-CC risk are likewise similar. When considering all-cause mortality, Black individuals have a 70% higher risk of PTC-CC than their counterpart White individuals. The SEER study trends in a similar direction, but not as drastically, with their finding of Black individuals having a 17.3% higher risk of PTC-CC [6]. Based on the SEER study, when compared with White patients, Black patients have a 17.3% higher risk of all-cause mortality following PTC-CC diagnosis; however, they actually have an 11.5% lower risk of thyroid-cancer-specific mortality [6].

Our overall 10-year survival rate for the columnar cell variant of papillary carcinoma is 81.6%, which is lower than the 93% survival rate for generalized papillary carcinomas reported in literature [4]. A point to note is that the 93% survival rate includes all papillary carcinomas, which encompass cancers ranging in aggressiveness. The lower survival rate of 81.6% for the columnar cell type may be explained due to its highly aggressive nature when comparing it with other papillary carcinomas [4].

Studies on the columnar cell variant of papillary thyroid carcinoma have limited sample sizes, with many ranging from one to 50 patients. This study analyzes PTC-CC with a relatively larger sample size of 7079 patients. The NCDB is fairly comprehensive, as it includes over 70% of official cancer diagnoses in the United States. However, patients diagnosed at non-Commission on Cancer (CoC)-accredited sites are not represented within this dataset. Thus, we are uncertain whether non-CoC patient data would be consistent with CoC patient data from NCDB. Additionally, since this study is retrospective in nature, the variables used in this study are limited to the NCDB; some variables have missing data, in which case the valid percentages were used. Lastly, NCDB reports overall survival and not cancer-specific survival. Interpretations of mortality rates may be skewed because PTC-CC-specific deaths and all-cause deaths are undifferentiated. Despite these limitations, many of our results are consistent with those published in the current literature and provide support to our conclusions.

## Conclusions

In this paper, we identified the demographic and prognostic factors associated with the overall survival of patients with the columnar cell variant of papillary thyroid carcinoma. Factors found to be positively correlated with mortality included age, comorbidities, and advanced cancer staging, especially in men and Black individuals. No differences in survival were found when comparing patients’ insurance status, education levels, or adjacent income quartiles. There were also no significant differences when comparing NCDB Analytic Stage II patients to Stage III patients or when comparing patients with a Charlson-Deyo score of 2 and above. Surgery represented the main treatment method, also used in combination with adjuvant radiation and adjuvant hormone therapies. Since PTC-CC typically metastasizes at varying rates and towards varying locations, future studies should focus on investigating varying metastasis profiles and their correlations with overall patient survival and treatment implications. As the understanding of this rare tumor continues to evolve, our conclusions contribute to the clinical knowledge surrounding the demographics and prognostics of the columnar cell variant of papillary thyroid carcinoma.
